# Parental risk factors associated with congenital heart disease in a Thai population: multivariable analysis

**DOI:** 10.2478/abm-2021-0033

**Published:** 2021-12-30

**Authors:** Chayamon Suwansumrit, Worawan Jittham

**Affiliations:** Department of Pediatrics, Faculty of Medicine, Naresuan University, Phitsanulok 65000, Thailand

**Keywords:** heart defects, congenital, heart disease risk factors, pregnancy, smoking, Thailand

## Abstract

**Background:**

Congenital heart diseases (CHDs) are the most common types of birth defects and contribute to a large proportion of infant morbidities and mortalities worldwide. These defects may require multiple surgical interventions impacting the infant's quality of life.

**Objectives:**

To identify risk factors associated with CHD in a population of Thai children.

**Methods:**

We conducted a case–control study of patients attending the Pediatric Clinic, Naresuan University Hospital, Thailand. We included data from pediatric patients diagnosed with CHDs as cases, and patients without cardiovascular abnormalities as controls. Risk data were collected from July 2019 to April 2020 using face-to-face interviews. Multiple logistic regression was used to analyze parental factors associated with CHDs.

**Results:**

We included 249 cases classified into 2 groups according to severity and 304 patients as controls. For those less-severely affected (155 patients, 62.2%), ventricular septal defect (27.7%) was the most prevalent, whereas for those with severe CHDs, tetralogy of Fallot was the most prevalent (14.0%). There was no difference in sex distribution or maternal obstetric history between the groups. In multivariable analysis, a family history of CHDs (adjusted odds ratio [AOR] 4.67, 95% confidence interval (CI) 1.61–13.57, *P* = 0.005) and maternal exposure to second-hand cigarette smoke (AOR 1.58, 95% CI 1.03–2.42, *P* = 0.002) were identified as significant risk factors for CHDs.

**Conclusion:**

A family history of CHDs and maternal exposure to second-hand cigarette smoke are associated with having offspring with CHDs in the population studied. These findings help us to encourage affected parents to obtain a fetal echocardiogram.

Among birth defects, congenital heart diseases (CHDs) contribute the largest proportion of all mortalities and morbidities, with a worldwide prevalence between 3.7 and 20 per 1,000 live births [1, 2, 3, 4, 5]. These defects may manifest in a variety of clinical presentations in a range of mild-to-fatal conditions for which some may need multiple surgical interventions, which can adversely affect both the infant and family's quality of life, leading to impaired physical capacities for the child, and increased family burden.

Morbidities and mortalities from CHDs vary regionally, in part, due to the differences in the availability of intensive medical or surgical care, as CHDs require multidisciplinary management and medical teams.

For the prenatal diagnosis of CHDs, a fetal echocardiogram was introduced decades ago and has become a widely used standard of routine screening for high-risk pregnancies [6]. Common indications for a fetal echocardiogram include suspicion of CHDs during standard obstetric screening, maternal diabetes mellitus (DM), mothers with family history of CHDs, and findings of extracardiac anomalies [7]. However, unrecognized CHDs remain with a prevalence of approximately 1 per 1,000 live births in rural Thailand [8, 9].

Risk factors that may be associated with having offspring with CHD have been the subject of intense investigation over the past decades. A large study in China found that advanced maternal age, maternal illnesses such as gestational diabetes mellitus (GDM) and pregnancy-induced hypertension (PIH), mothers who have family history of CHDs, and lower socioeconomic status are associated with bearing children with CHD [2].

Many studies have found that maternal smoking increases the chances of having offspring with CHD, but for maternal exposure to second-hand cigarette smoke, the findings are inconsistent [10, 11, 12, 13, 14, 15, 16]. Some studies found no association between maternal alcohol intake and fetal CHDs [12, 17, 18], while more recent studies found significant associations [10, 13, 16]. Other environmental hazards found to be associated with fetal CHDs include exposure to pesticides, chemicals, organic solvents, or paint, and a history of living in a renovated house [16, 19, 20, 21, 22, 23, 24, 25, 26].

Here, we sought to identify parental risk factors associated with CHDs in a population from a mostly rural province in Thailand using multivariable analysis to control for confounding factors. The results were anticipated to support primary prevention strategies, and to provide additional fetal screening, which would enhance postnatal management and long-term patient care.

## Materials and methods

**Figure 1:**
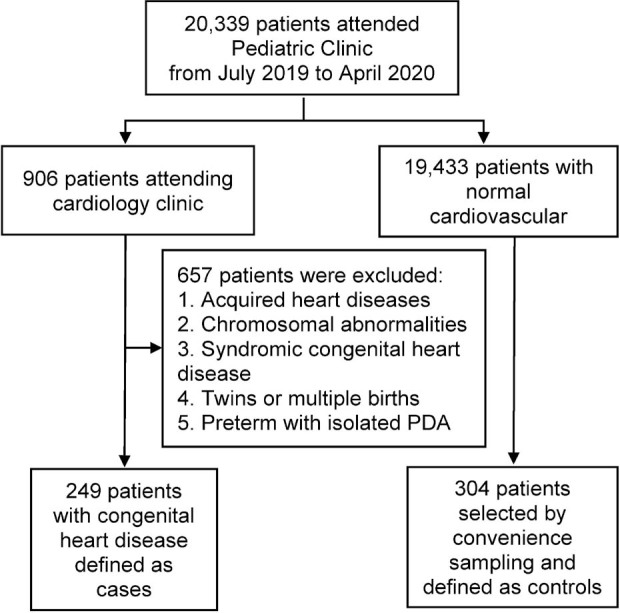
Participant flow diagram of CHD cases and patient controls. CHD, congenital heart diseases; PDA, patent ductus arteriosus.

This case–control study was conducted in the Pediatric Clinic, Department of Pediatrics, Naresuan University Hospital, a 400-bed tertiary care medical center and university teaching hospital in the mostly rural lower northern region of Thailand. Ethics approval for this study was obtained from Naresuan University Institutional Review Board (IRB No. 0106/62; certificate of approval No. 215/2019, June 14), and followed the principles of the Declaration of Helsinki and its contemporary revisions, recommendations of The Belmont Report, the guidelines of the Council for International Organizations of Medical Sciences, and International Conference on Harmonization in Good Clinical Practice. Transparent reporting of a multivariable prediction model for individual prognosis or diagnosis (TRIPOD) [27] was used for this article. We included data related to 553 case and control patients attending the Clinic from July 2019 to April 2020. We conducted a retrospective review of medical records to include 249 patients with CHD diagnosis confirmed by echocardiography as cases; and 304 patients without cardiovascular abnormalities, as examined by pediatricians who visited at the Pediatric Clinic during the time of the study, were selected by convenience sampling and defined as controls. Patients with acquired heart diseases, chromosomal abnormalities, syndromic CHD, twins or multiple birth, and preterm birth with isolated patent ductus arteriosus (PDA) were excluded **(Figure 1)**.

The CHDs cases were classified into 2 groups: severe CHDs and less-severe CHDs. Severe CHDs were defined as complex lesions, for example, hypoplastic left heart syndrome (HLHS), single ventricle, tricuspid valve atresia, truncus arteriosus, interrupted aortic arch, pulmonary atresia without ventricular septal defect (VSD), d-transposition of the great arteries, double outlet right ventricle (DORV), atrioventricular canal defects, total anomalous pulmonary venous return (TAPVR), tetralogy of Fallot (TOF), and Ebstein anomaly. By contrast, less-severe CHDs were defined as any single cardiac lesions, which include VSD, atrial septal defect (ASD), coarctation of the aorta (COA), PDA, and any valvular stenosis or regurgitation.

Parents of all participants were counseled and informed consent was obtained for their data and those of their child to be included in a study. Patients’ demographic data (sex, maternal obstetric history, family history of CHD) and parental risk factors during pregnancy including parental age, gestational complications, parental substance use (smoking and alcohol drinking), history of maternal exposure to second-hand smoke, history of environmental contaminant exposure (pesticides, chemicals, or paint), and socioeconomic status (maternal educational attainment level, occupation and family income) were collected during face-to-face interviews conducted by medical personnel.

Demographic data related to cases and controls were compared using χ^2^ and Fisher exact tests. Univariate analysis was used to determine parental risk factors for CHDs with odds ratio (OR) and 95% confidence interval (CI). We conducted multivariable analysis of all significant univariate risk factors including the patient's age, sex, maternal GDM, positive family history of CHDs, maternal exposure to second-hand smoke, paternal smoking, paternal alcohol drinking, history of pesticide exposure, maternal occupation, maternal educational attainment level, and family income. The factors are presented in terms of adjusted odds ratio (AOR) and 95% CI. *P* < 0.05 was considered as significant, and the risk comparison among cases was classified according to severity. All statistical calculations were performed using Stata version 14 for Windows (StataCorp).

We sought to determine risk factors for CHD in our population. The sample size was calculated based on the effect of maternal smoking (OR = 2.0) by Fung et al. [10] with the prevalence of maternal smoking of 10%, α of 0.05, and β of 0.80; so, the predicted sample size for case and control was 295 in each case. Maternal smoking is a high OR risk factor and there was a high prevalence of smoking in the population. We also calculated sample size for other risk factors with a smaller effect size. However, the estimated sample size was so high that we could not achieve it. We decided to use one case per one control. However, due to a limitation of cases in our hospital and management, we ultimately had a sample size of 249 for cases, 304 for controls, and we considered that it would not decrease the validity of the present study.

## Results

There was no significant difference in maternal obstetric history (gravida, parity, history of abortion, or gestational age) between case and control groups **(Table 1)**.

**Table 1:** Demographic data for cases of CHD and unaffected control patients.

**Characteristic**	**Case (n = 249)** **n (%)**	**Control (n = 304)** **n (%)**	***P***
Sex^†^			0.033
Male	124 (49.8)	179 (58.9)	
Female	125 (50.2)	125 (41.1)	
Age^†^ (years)			<0.001
<1	50 (20.1)	71 (23.4)	
1–5	86 (34.5)	165 (54.2)	
6–10	63 (25.3)	47 (15.5)	
>10	50 (20.1)	21 (6.9)	
Gravida			0.29
Primigravida	112 (45.0)	123 (40.5)	
Multigravida	137 (55.0)	181 (59.5)	
Parity			0.67
1	118 (47.4)	140 (46.0)	
2	101 (40.6)	133 (43.8)	
3 or more	30 (12.0)	31 (10.2)	
History of abortion^‡^			0.050
None	220 (88.4)	252 (82.9)	
1	27 (10.8)	41 (13.5)	
2 or more	2 (0.8)	11 (3.6)	
Gestational weeks^‡^			0.02
<34	0 (0)	8 (2.6)	
34–36	26 (10.4)	36 (11.8)	
≥ 37	223 (89.6)	260 (85.5)	

† χ^2^ test

‡ Fisher exact test.

CHD, congenital heart disease.

In patients with CHDs, 155 (62.2%) of the 249 cases were diagnosed as less-severe CHD and the remainder as severe CHD. Of all cases, VSD was the most frequent CHD in the present study (69 cases; 27.7%), followed in order by ASD, PDA, pulmonary stenosis, aortic stenosis, aortic regurgitation, and COA. Among those with severe CHD, TOF was the most frequent diagnosis (35 cases; 14.0%), followed by DORV, single ventricle, and pulmonary atresia with VSD (PA/VSD), respectively **(Table 2)**.

**Table 2:** Diagnosis of congenital heart disease.

**Diagnosis**	**n = 249 (%)**
**Less severe**	**155 (62.2**)
VSD	69 (27.7)
ASD	41 (16.5)
PDA	22 (8.8)
Pulmonary stenosis	20 (8.0)
Aortic stenosis	1 (0.4)
Aortic regurgitation	1 (0.4)
Coarctation of aorta	1 (0.4)
**Severe**	**94 (37.8)**
TOF	35 (14.0)
Double outlet of right ventricle	13 (5.2)
Single ventricle	11 (4.4)
Pulmonary atresia with VSD	8 (3.2)
Ebstein anomaly	7 (2.8)
Transposition of the great vessels	7 (2.8)
Pulmonary atresia with intact ventricular septum	4 (1.6)
Atrioventricular canal defect	4 (1.6)
Tricuspid atresia	3 (1.2)
Anomalous left coronary artery from the pulmonary artery	1 (0.4)
TAPVR	1 (0.4)

ASD, atrial septal defect; PDA, patent ductus arteriosus; TOF, tetralogy of Fallot; TAPVR, total anomalous pulmonary venous return; VSD, ventricular septal defect.

Parental risk factors were determined by univariate logistic regression. Among the familial biological data, we found that mothers with reported family history of any CHD were at risk of having children with CHD (OR 4.58, 95% CI 1.82–11.52, *P* = 0.001), but we did not find any significant association with parental age **(Table 3)**.

**Table 3:** Familial biological data as a risk of CHDs on univariate analysis.

**Characteristic**	**Case (n = 249)** **n (%)**	**Control (n = 304)** **n (%)**	**OR**	**95% CI**	***P***
**Family history of congenital heart disease**					0.001^*^
Yes	21 (8.4)	6 (2.0)	4.58	1.82–11.52	
No	228 (91.6)	298 (98.0)	1.00	Ref.	
**Maternal age (years)**					0.67
<20	26 (10.4)	27 (8.9)	1.00	Ref.	
20–35	183 (73.5)	221 (72.7)	0.86	0.49–1.53	
≥ 35	40 (16.1)	56 (18.4)	0.74	0.38–1.46	
**Paternal age (years)**					0.84
<20	11 (4.4)	16 (5.3)	1.00	Ref.	
20–35	164 (65.9)	194 (63.8)	1.23	0.56–2.72	
≥ 35	74 (29.7)	94 (30.9)	1.15	0.50–2.62	
**Maternal pregnancy complication**					
**GDM**					0.013^*^
Yes	11 (4.4)	31 (10.2)	0.41	0.20–0.83	
No	238 (95.6)	273 (89.8)	1.00	Ref.	
**Pregnancy-induced hypertension**					0.11
Yes	8 (3.2)	19 (6.3)	0.50	0.21–1.16	
No	241 (96.8)	285 (93.7)	1.00	Ref.	

* *P* < 0.05, significant.

CHDs, congenital heart diseases; CI, confidence interval; GDM, gestational DM; Ref. reference; OR, odds ratio.

Significant hazardous exposure risks for parents having children with CHDs as found by univariate analysis were maternal exposure to second-hand smoke (OR 2.33, 95% CI 1.65–3.28, *P* < 0.001), paternal smoking (OR 1.69, 95% CI 1.21–2.37, *P* = 0.002), and history of pesticide exposure (OR 3.63, 95% CI 2.16–6.09, *P* < 0.001) **(Table 4)**.

**Table 4:** Parental environmental hazard exposure on univariate analysis.

**Characteristics**	**Case (n = 249)** **n (%)**	**Control (n = 304)** **n (%)**	**OR**	**95% CI**	***P***
**Maternal smoking**					0.11
Yes	6 (2.4)	2 (0.7)	3.73	0.75–18.64	
No	243 (97.6)	302 (99.3)	1.00	Ref.	
**Maternal exposure to second-hand smoke**					<0.001^*^
Yes	151 (60.6)	121 (39.8)	2.33	1.65–3.28	
No	98 (39.4)	183 (60.2)	1.00	Ref.	
**Paternal smoking**					0.002^*^
Yes	134 (53.8)	124 (40.8)	1.69	1.21–2.37	
No	115 (46.2)	180 (59.2)	1.00	Ref.	
**Maternal alcohol drinking**					0.98
Yes	16 (6.5)	20 (6.6)	0.98	0.50–1.93	
No	232 (93.5)	284 (93.4)	1.00	Ref.	
**Paternal alcohol drinking**					0.036^*^
Yes	176 (70.7)	189 (62.2)	1.47	1.03–2.10	
No	73 (29.3)	115 (37.8)	1.00	Ref.	
**History of pesticide exposure**					<0.001^*^
Yes	57 (22.9)	23 (7.6)	3.63	2.16–6.09	
No	192 (77.1)	281 (92.4)	1.00	Ref.	
**History of chemical exposure**					0.53
Yes	5 (2.0)	4 (1.3)	1.54	0.41–5.79	
No	244 (98.0)	300 (98.7)	1.00	Ref.	
**History of paint exposure**					0.38
Yes	12 (4.8)	20 (6.6)	0.72	0.34–1.50	
No	237 (95.2)	284 (93.4)	1.00	Ref.	

* *P* < 0.05; Ref. reference; Case, case of congenital heart disease; Control, patient control.

CI, confidence interval; OR, odds ratio.

The univariate analysis found parental socioeconomic status factors that reduce the risk of having children with a CHD included maternal GDM (OR 0.41, 95% CI 0.20–0.83, *P* = 0.013), higher educational attainment by the mother (OR 0.79, 95% CI 0.20–0.83, *P* < 0.001), and higher family income (OR 0.88, CI 0.56–1.39, *P* < 0.001) **(Table 5)**.

**Table 5:** Parental socioeconomic status univariate analysis.

**Characteristic**	**Case (n = 249)** **n (%)**	**Control (n = 304)** **n (%)**	**OR**	**95% CI**	***P***
**Occupation**					<0.001^*^
Farmer	38 (15.3)	9 (3.0)	2.35	0.63–8.72	
Manual worker	33 (13.3)	11 (3.6)	1.67	0.46–6.05	
Merchant/self-employed	30 (12.0)	42 (13.8)	0.40	0.12–1.30	
Employee	74 (29.7)	80 (26.3)	0.51	0.17–1.60	
Government officer	22 (8.8)	90 (29.7)	0.14	0.04–0.45	
Housekeeper	43 (17.3)	67 (22.0)	0.36	0.114–1.14	
Student	9 (3.6)	5 (1.6)	1.00	Ref.	
**Maternal educational attainment**					<0.001^*^
Primary school or lower	52 (20.9)	27 (8.9)	1.00	Ref.	
Junior high school	56 (22.5)	37 (12.2)	0.79	0.42–1.47	
High school	87 (34.9)	92 (30.3)	0.49	0.28–0.85	
Bachelor degree or higher	54 (21.7)	148 (48.6)	0.19	0.11–0.33	
**Family income per month baht (USD)^†^**					<0.001^*^
<10,000 (<322)	55 (22.1)	44 (14.5)	1.00	Ref.	
10,000–30,000 (322–966)	152 (61.1)	138 (45.4)	0.88	0.56–1.39	
30,001–50,000 (966–1611)	25 (10.0)	76 (25.0)	0.26	0.14–0.48	
>50,000 (>1611)	17 (6.8)	46 (15.1)	0.30	0.15–0.59	

Case, patient with congenital heart disease; Control, patient control.

* *P* < 0.05.

† U.S. Federal Reserve G.5A annual foreign exchange rates for 2019 available from: https://www.federalreserve.gov/releases/g5a/current/

CI, confidence interval; OR, odds ratio; SES, socioeconomic status.

In the multivariable analysis of all significant univariate risk factors—patient's age, sex, maternal GDM, positive family history of CHDs, maternal exposure to second-hand smoke, paternal smoking, paternal alcohol drinking, history of pesticide exposure, maternal occupation, maternal educational attainment, and family income—there were 2 factors, positive family history of CHDs (AOR 4.67, 95% CI 1.61–13.57, *P* = 0.005) and maternal exposure to second-hand smoke exposure (AOR 1.58, 95% CI 1.03–2.42, *P* = 0.002), which were significantly associated with increase the risk of having children with a CHD **(Table 6)**. We found that mothers with a reported family history of CHDs are more likely to bear children with a severe form of CHDs than those who did not. Moreover, mothers who were exposed to second-hand smoke are more likely to bear children with a less-severe form of CHD.

**Table 6:** Parental risk factors for CHDs on multiple logistic regression.

**Factor**	**Case (n = 249)** **n (%)**	**Control (n = 304)** **n (%)**	**AOR**	**95% CI**	***P***
**Family history of congenital heart disease**					0.005^*^
Yes	21 (8.4)	6 (2.0)	4.67	1.61–13.57	
No	228 (91.6)	298 (98.0)	1.00	Ref.	
**Maternal exposure to second-hand smoke**					0.002^*^
Yes	151 (60.6)	121 (39.8)	1.58	1.03–2.42	
No	98 (39.4)	183 (60.2)	1.00	Ref.	
**GDM**					
Yes	11 (4.4)	31 (10.2)	0.60	0.27–1.33	0.21
No	238 (95.6)	273 (89.8)	1.00	Ref.	
**Paternal smoking**					0.12
Yes	134 (53.8)	124 (40.8)	1.45	0.23–2.88	
No	115 (46.2)	180 (59.2)	1.00	Ref.	
**Paternal alcohol drinking**					0.16
Yes	176 (70.7)	189 (62.2)	1.38	0.88–2.16	
No	73 (29.3)	115 (37.8)	1.00	Ref.	
**History of pesticide exposure**					0.11
Yes	57 (22.9)	23 (7.6)	1.88	0.87–4.05	
No	192 (77.1)	281 (92.4)	1.00	Ref.	
**Occupation**					0.64
Farmer	38 (15.3)	9 (3.0)	0.68	0.14–3.32	
Manual worker	33 (13.3)	11 (3.6)	1.32	0.32–5.50	
Merchant/Self-employed	30 (12.0)	42 (13.8)	0.32	0.09–1.22	
Employee	74 (29.7)	80 (26.3)	0.43	0.18–1.56	
Government officer	22 (8.8)	90 (29.7)	0.16	0.04–0.64	
Housekeeper	43 (17.3)	67 (22.0)	0.24	0.07–0.89	
Student	9 (3.6)	5 (1.6)	1.00	Ref.	
**Maternal educational attainment**					0.50
Primary school or lower	52 (20.9)	27 (8.9)	1.00	Ref.	
Junior high school	56 (22.5)	37 (12.2)	1.58	0.71–3.56	
High school	87 (34.9)	92 (30.3)	1.59	0.78–3.26	
Bachelor degree or higher	54 (21.7)	148 (48.6)	1.13	0.63–2.03	
**Family income per month baht (USD)^†^**					0.53
<10,000 (323)	55 (22.1)	44 (14.5)	1.00	Ref.	
10,000–30,000 (323–996) USD)	152 (61.1)	138 (45.4)	1.08	0.43–2.70	
30,001–50,000 (996–1610)	25 (10.0)	76 (25.0)	1.40	0.65–3.00	
>50,000 baht (>1610)	17 (6.8)	46 (15.1)	0.96	0.43–2.12	

* *P* < 0.05.

† U.S. Federal Reserve G.5A annual foreign exchange rates for 2019 available from: https://www.federalreserve.gov/releases/g5a/current/

AOR, adjusted odds ratio; CHDs, congenital heart diseases; CI, confidence interval; GDM, gestational DM.

## Discussion

The family history of CHD is a crucial risk factor, and has been recognized for decades as an indication for fetal echocardiography. The present study showed significant association of family history, in both univariate and multivariable analysis of our population (AOR 4.67, 95% CI 1.61–13.57, *P* = 0.005), as consistent with findings by Fung et al. [10], who reported that mothers who reported 3 generations of CHD are at 2-fold higher risk of having a baby with the same condition. Several studies found a similar correlation for overall CHDs [2, 11, 28]. Roodpeyma et al. [29] reported that a history of CHD in siblings was associated with CHD in subsequent children. This correlation indicates that genetics play a role in the occurrence of CHD. The present study also found that family history was a risk factor strongly related to severity of the congenital heart lesion.

Several studies showed that maternal age was associated with a CHD in their infant. Liu X et al. [2], Bassili et al. [28], and Hollier et al. [30] found that mothers aged ≥40 years are at risk of having a child with CHD. Similarly, Reefhuis et al. [31], Miller et al. [32], and Malik et al. [15] reported that the maternal age for risk is ≥35 years. Advanced maternal age, especially >35 years old, is associated with many types of birth defects, including Down syndrome, which strongly indicate prenatal chromosomal analysis in these pregnancies. Even though chromosomal defects were excluded from the studies mentioned above, advanced maternal age was still significantly correlated with having a child with CHD. Younger maternal age is associated with some types of heart defects including TAPVR and tricuspid valve atresia [12]. The link between paternal age and the risk of having offspring with CHD has not been studied thoroughly, and findings remain inconsistent thus far.

Materna-Kiryluk et al. found that advanced paternal age is associated with increased risk of CHD [33], whereas Patel and Burns [12] reported the relationship of paternal age and offspring with CHD as a U-shaped curve, reflecting that both younger and older age of fathers are at risk. However, the present study found no association related to the age of parents and children born with CHD. Further study with larger sample sizes is recommended to determine any association.

Maternal DM has been found associated with CHDs by numerous investigators. Embryonic hyperglycemia potentially plays an important role because of abnormal glucose levels in diabetic patients that disrupt fetal metabolism and the expression of regulatory genes in the embryo that may alter organ structure development [5, 21]. A prevalence of 318 per 10,000 live births compared with a baseline risk of 80 per 10,000 children born with CHD in mothers with pregestational DM has been reported [14]. Mothers with known DM complications were found to have a higher risk of having offspring with CHD than those who have no complications. In addition, GDM is associated with a lower risk of having offspring with CHD than pregestational DM [5]. Mothers with type 1 DM have an increased risk of having children with CHD in contrast to mothers with GDM [10]. We found an association with decreased risk of having a child with heart defects and maternal GDM in univariate analysis, but not with an adjusted result in multivariable analysis.

Some studies have found that mothers who are hypertensive during pregnancy have a higher risk of bearing a child with a heart defect [2, 12]. We found no association with pregnancy-induced hypertension and CHD, as consistent with the findings in the large population study in China by Fung et al. [10]. The actual biologically related mechanism of hypertension and fetal cardiac anomaly remains unclear.

The association of maternal cigarette smoking, among smoking other hazardous substances, with congenital anomalies has been investigated frequently. Woods and Raju [22], Li et al. [23], and Lee and Lupo [24] found a strong association with about 44%–56% increased risk of developing CHDs, such as right ventricular outflow tract obstruction, pulmonary valve stenosis, and VSD in smoking mothers. Malik et al. [15] found that maternal smoking was associated with septal defects, but found no significant relationship in passive smokers. However, other studies have reported that the relationship between maternal smoking or maternal passive smoke exposure and CHD is inconsistent [12, 17]. We found no significant association between maternal smoking and CHDs (AOR = 3.73, 95% CI 0.75–18.64, *P* = 0.11). This may be related to the lack of power from the small number of smoking mothers among the respondents in our study. Nevertheless, we found strong association between maternal exposure to second-hand smoke and CHDs (AOR 1.58, 95% CI 1.03–2.42, *P* = 0.002). Univariate analysis showed paternal smoking associated significantly with CHD, but not in multivariable analysis. We concluded that the reason paternal smoking was not found significantly associated with CHD might be that paternal smoking and maternal exposure to second-hand smoke were associated with each other and collinear.

Early studies describe various mechanisms of smoking and how it restricts fetal development. Nicotine and carbon monoxide are highly damaging components in tobacco smoke that can cross the placenta, inducing vasoconstriction leading to fetal hypoxia. Moreover, nicotine also inhibits the expression of cardiac differentiation genes and depresses early cardiac development [23]. It is likely that gene-to-gene, gene-to-environment, or environment-to-environment connections play a considerable role in the congenital defects, as has been studied widely. The gene for glutathione S-transferase (*GST*) is reported to be associated with CHD anomalies. *GST* is a part of polymorph supergene family that is involved in detoxification and metabolism of several toxins, and helps modulate their adverse effects. There are 4 main classes of GST; A, M, P, and T, in which GST-M and GST-T are mostly researched. Li et al. [23] studied modification of the association between maternal smoke exposure and CHD. They found that mothers exposed to second-hand smoke who had any functional deletion on *GST*, either GST-M or GST-T, had a higher risk of having a child with CHD. This represents the important role of gene–environment interaction as mothers with *GST* variants showed a lower threshold to having a child with CHD compared with those without the variation despite equal smoke exposure.

Ethanol plays a role in impairment of fetal structural heart formation as it causes fetal tissue edema and affects the primitive cardiac loop [21]. Fung et al. [10] and Liu et al. [16] found that maternal alcohol intake significantly increases the risk of having a child with a CHD, while other investigations found no association [12, 16]. We found no association between either maternal or paternal alcohol consumption and CHD.

Pesticides have been widely used in many countries including Thailand, particularly in rural regions. Of potential environmental factors, pesticides are the most widely studied. Most commercially used pesticides contain several chemical components including hydrocarbons; these harmful substances can persist in the environment due to their resistance to degradation. Exposure to pesticides, especially during the critical period of cardiovascular development, can the increase risk of having a child with some type of cardiac defect such as VSD or TGA [26]. A significant association has been found between fetal CHD and environmental hazards including pesticides and organic solvents [20]. Although we found significant association between pesticides and offspring with CHD in univariate logistic regression, in multivariable analysis the association between maternal pesticide exposure and risk for an offspring with CHD was not significant. Additional studies of molecular correlation are probably helpful to investigate the relationship, and further, provide information for primary prevention policy.

Organic solvents, including ethanol, benzene, and byproducts of the metal industry, are also hazardous environmental factors, and several studies have found an association between organic chemicals and CHD in infants with unknown mechanisms. Patel et al. [12] reported a correlation between organic solvents and the incidence of COA, HLHS, and TGA. This finding is supported by those of others who found that organic solvents and other chemicals are associated with cases of CHD overall [13, 16, 25]. However, we found no significant association of CHD with organic solvents, possibly because of the small number of parents included who were exposed to the solvents.

Previous studies have found an association between lower socioeconomic status, including lower educational attainment levels or family incomes, and having a child with CHD [2, 12, 14]. By contrast, the present study found no association between socioeconomic status of mothers in multivariable analysis.

To our knowledge, this is the first study of parental risk factors for CHDs conducted in rural Thailand. The results may contribute toward improving primary prevention and family planning strategies for modifiable risk factors, and might reflect associations in other regions with similar ethnic, environmental, and agrarian backgrounds. Nevertheless, there are some limitations to the present study. There is possibly a recall bias as interviews were conducted to determine factors. A lack of statistical power might explain why some factors that were significantly associated with incidence of CHDs in the univariate analysis were not significant in the multivariable analysis. The sample size calculation did not apply to all factors significant in the univariate analysis. As the participants included a control group of those who visited the pediatric outpatient clinic for routine vaccination as the common reason of visiting, the age and sex of the participants in the groups may not be matched exactly. Although this was a single-center study representing a specific region in Thailand, we found factors significantly associated with CHD that are consistent with studies using larger sample size from other countries. Further investigation of genetic or molecular correlations may be helpful for primary prevention strategies and larger multicenter studies in Thailand are warranted to identify further risks in the Thai population.

## Conclusion

Our study highlights the increasing problem of associated risks for bearing children with CHDs. Exposure to second-hand cigarette smoke and a family history of CHD are the parental risk factors most strongly associated with bearing children with CHDs. Maternal exposure to second-hand smoke is a risk factor that can be modified to avoid CHD during embryogenesis. Family history of CHD is nonmodifiable, but consideration should be given to antenatal screening for early detection. These findings support encouraging affected parents to request a fetal echocardiogram.
